# Highly efficient MoO_x_-free semitransparent perovskite cell for 4 T tandem application improving the efficiency of commercially-available Al-BSF silicon

**DOI:** 10.1038/s41598-018-34432-5

**Published:** 2018-10-31

**Authors:** F. Javier Ramos, Sebastien Jutteau, Jorge Posada, Adrien Bercegol, Amelle Rebai, Thomas Guillemot, Romain Bodeux, Nathanaelle Schneider, Nicolas Loones, Daniel Ory, Cedric Broussillou, Gilles Goaer, Laurent Lombez, Jean Rousset

**Affiliations:** 1 IPVF, Ile-de-France Photovoltaic Institute (IPVF), 30 Route Départementale 128, 91120 Palaiseau, France; 2 CNRS, Ile-de-France Photovoltaic Institute (IPVF), UMR 9006, 30 route départementale 128, 91120 Palaiseau, France; 3 EDF R&D, 30 Route Départementale 128, 91120 Palaiseau, France; 4 Licorne Laboratory, ECE Paris, 37 quai de Grenelle, 75015 Paris, France; 5 Photowatt, EDF ENR PWT, 33 rue Saint-Honoré, Z.I. Champfleuri, 38300 Bourgoin-Jallieu, France

## Abstract

In this work, the fabrication of MoO_x_-free semitransparent perovskite solar cells (PSC) with Power Conversion Efficiencies (PCE) up to 15.7% is reported. Firstly, opaque PSCs up to 19.7% were fabricated. Then, the rear metal contact was replaced by a highly transparent and conductive indium tin oxide (ITO) film, directly sputtered onto the hole selective layer, without any protective layer between Spiro-OMeTAD and rear ITO. To the best of our knowledge, this corresponds to the most efficient buffer layer-free semitransparent PSC ever reported. Using time-resolved photoluminescence (TRPL) technique on both sides of the semitransparent PSC, Spiro-OMeTAD/perovskite and perovskite/TiO_2_ interfaces were compared, confirming the great quality of Spiro-OMeTAD/perovskite interface, even after damage-less ITO sputtering, where degradation phenomena result less important than for perovskite/TiO_2_ one. Finally, a 4-terminal tandem was built combining semitransparent PSC with a commercially-available Aluminium Back Surface Field (Al-BSF) silicon wafer. That silicon wafer presents PCE = 19.52% (18.53% after being reduced to cell size), and 5.75% once filtered, to generate an overall 4 T tandem efficiency of 21.18% in combination with our champion large semitransparent PSC of 15.43%. It means an absolute increase of 1.66% over the original silicon wafer efficiency and a 2.65% over the cut Si cell.

## Introduction

In the last five years, hybrid organic-inorganic perovskite solar cells (PSC) have emerged as one of the most promising photovoltaic (PV) technologies. This remarkable development is principally related to the high absorption coefficient and carrier diffusion lengths larger than the film thickness needed to absorb the solar spectrum^1–3^. With a careful growth of perovskite film, structural and optoelectronic properties have been successfully controlled, permitting to obtain power conversion efficiencies (PCE) over 22% at lab scale^4^. Moreover, these materials showed an easily tunable bandgap by modifying its composition^5–7^. This property, together with a remarkably low sub-bandgap absorption make perovskite materials interesting candidates for top cell absorbers in tandem devices^8^. Generally speaking, a tandem solar cell is composed by a stack of two or more subcells with bandgaps decreasing from top to bottom device, where the most energetic part of the solar spectrum (short wavelengths) is absorbed by the top cell while larger wavelengths are collected by the bottom one. Recently, PCE up to 26.4% have been achieved by the combination of a perovskite top cell with a crystalline silicon bottom one^9–14^. For the production of a tandem solar cell with two absorbers, two main connection approaches are proposed in the literature: 2-terminal (2 T) and 4-terminal (4 T) geometries, presenting each one their own advantages and weaknesses.

For 2 T geometry, carriers from the top cell have to recombine with those ones with opposite charge coming from the bottom one at the tunnel junction, without creating too high electrical or optical losses. For this purpose, indium tin oxide (ITO) has been the most explored material in literature leading to efficiencies up to 23.6% for perovskite/Si 2 T tandem cells^11^. Moreover, in the case of 2 T perovskite/Si tandem geometry, a careful current matching between top and bottom cell is essential, since the overall tandem efficiency resulted extremely sensitive to photocurrent fluctuations between top and bottom cell^15,16^. The required 2 T current matching can be achieved for a fixed AM1.5 G spectra either by tuning the perovskite bandgap^6^ up to an ideal value of ~1.75 eV^17,18^ or by reducing the thickness of the perovskite absorber, allowing certain wavelengths (normally between 650–800 nm) to pass through perovskite and being absorbed by the silicon underneath. The former option is reasonably feasible due to tunable bandgap of perovskite^19^, while the latter one seems to be harder to accomplish since the extremely high absorption coefficient of perovskite^20^ would force to control the thickness of absorber film with an exceptionally high precision. Anyway, independently from the current-matching approach chosen, it would be optimized for a particular spectrum (normally AM1.5 G at 1 sun intensity) and the global tandem PCE would be significantly reduced when the incidence spectrum was varied due to daytime or seasonal changes.

On the contrary, recent simulation studies have also demonstrated a maximum PCE for a 4 T perovskite/Si tandem cell with a large band gap perovskite absorber *ca*. 1.75 eV^16^. However, in this case, the decrease of the performance related to spectral changes and current-mismatching resulted poorly important in comparison with 2 T configuration^16^. Moreover, with this tandem architecture, a semitransparent PSC can be mechanically stacked in an easy manner over a silicon solar cell or panel without any change in the production process and being applicable for any type of bottom cell architecture. For example, 4 T Tandem devices have been built with amorphous silicon/crystalline silicon heterojunction (SHJ)^9^ and Interdigitated Back Contact (IBC) silicon solar cell^14^ as bottom cell. However, for 4 T tandem cells, the introduction of a transparent conductive oxide (TCO) as rear electrode in the semitransparent perovskite top cell is required to be wisely optimized in order to minimize parasitic optical losses, mainly in the near infrared region (NIR) due to free-carrier absorption^21,22^.

Here, we report the association of a semitransparent PSC with a silicon bottom one for the assembly of a 4 T tandem device. Silicon material on bottom consisted on a commercially-available Aluminium Back Surface Field (Al-BSF) silicon wafer (PCE = 19.52%), which is the current dominant installed PV technology, conveniently reduced to the desired size by laser cutting (18.53% of PCE after cutting). Regarding the preparation of semitransparent top perovskite cell, the first step was the fabrication of a highly efficient opaque PSC (PCE = 19.7%) to be used as reference, followed by the selection of the most convenient sputtered-ITO properties as a trade-off between transmission and resistivity. For semitransparent PSC fabrication, to prevent any additional fabrication step, ITO layer was directly deposited onto Spiro-OMeTAD by radio frequency (RF) magnetron sputtering without any extra protection layer such as the usually reported molybdenum oxide (MoO_x_)^22,23^. It is noteworthy that the fabrication of semitransparent PSC without buffer layer between extraction layer and rear TCO has been a topic not sufficiently explored in literature, since damage issues on the substrate linked to the highly energetic nature of sputtering technique are difficult to circumvent. One notable exception was the work reported by Jaysankar *et al*. where ITO was directly sputtered onto Spiro-OMeTAD by direct current (DC) magnetron sputtering^24^. However, the efficiencies obtained remained not too high for semitransparent PSC (14.4% for 0.13 cm^2^ devices and 12% for 4 cm^2^ ones), with a significant reduction of *J*_*SC*_ during upscaling. Wahl *et al*., proposed indium zinc oxide (IZO), as semitransparent rear contact deposited onto bathocuproine/[6,6]-Phenyl-C_61_-butyric acid methyl ester (BCP/PCBM) with an inverted semitransparent PSC configuration improving the transmission in the infrared part of the spectra (800–1300 nm) and getting 13.3% also using a DC system^25^. Although the employment of RF magnetron sputtering for the fabrication of buffer layer-free semitransparent PSCs remained unexplored, RF technique has been widely reported in PSCs not only to grow different carrier transport layers such as TiO_2_^26^ or SnO_2_^27^, but also to prepare a PbS that can be easily transformed into PbI_2_ for the fabrication of the perovskite layer^28^. Moreover, RF technique showed certain advantages over DC for ITO deposition, since lower resistivities, higher mobilities and larger grain sizes were achieved for oxygen-free ITO sputtering at low temperature^29,30^. Furthermore, ITO-sputtered films were demonstrated to enable long-term and thermal stability for perovskites in tandem applications^31^, diminishing a concern that has focused important efforts for the research community^32,33^.

Hence, by introducing a RF sputtering and controlling the annealing conditions of ITO after deposition, we were able to control with precision ITO properties and to avoid any damage both in Spiro-OMeTAD and in the rest of the PSC, achieving a maximum PCE of 15.7% for small (0.16 cm^2^ active area) and 15.43% for larger (0.64 cm^2^ active area) semitransparent PSC with good absorption in UV-Vis and transmittance in NIR. Up to our knowledge, these results imply the best performing buffer-free semitransparent PSC ever reported for tandem purposes. By bifacial time-resolved photoluminescence (TRPL) Spiro-OMeTAD/perovskite and perovskite/TiO_2_ interfaces were compared. Using a numerical model^34^, optoelectronic properties of the bulk perovskite as well as interfaces were analysed to evaluate diffusion and recombination phenomena in the device^35^, revealing the great quality of Spiro-OMeTAD/perovskite interface where degradation phenomena are less marked than for perovskite/TiO_2_ one. Finally, a 4 T tandem device was prepared, reaching a remarkable efficiency of 21.18% that corresponds to a maximum absolute efficiency improvement of 1.66% of the commercially-available silicon Al-BSF wafer here employed, and a 2.65% boost when is compared to the Si cell once cut.

## Results and Discussion

### Optimization of a high efficient opaque perovskite solar cell

The first part of this study is focused on the growth of high quality perovskite films and particularly on the influence of the caesium addition in the perovskite formula. Three different perovskite absorbers with general formula Cs_x_(MA_0.166_FA_0.833_)_1-x_Pb(Br_0.166_I_0.833_)_3_ were prepared, where *x* = 0, 0.05 and 0.10 and they were coded as 0%, 5% and 10% Cs respectively. Top view Scanning Electron Microscopy (SEM) images of these perovskite layers are presented in Fig. S1 (Supporting information). As the impact of caesium on grain size distribution was not obvious, SEM images were analysed using *ImageJ* software in order to determine the surface occupied by each grain. The grain size distribution for the different perovskites prepared is shown in the frequency histograms (Fig. S1). Two main effects are evidenced: as Cs content was increased, the grain size distribution was narrower and the mean value was decreased from 190 nm (0% Cs) to 155 nm (10% Cs). Consistently, the crystallite size evaluated with the main diffraction peak of the perovskite phase located at 2*θ* ≈ 14° on the X-ray diffraction (XRD) patterns (Fig. S2) using the Debye-Scherrer formula also decreases with the addition of caesium (123 nm for 0% Cs, 114 nm for 5% Cs and 80 nm for 10% Cs). These results seem to be caused by a faster crystallization, in agreement with the findings reported by Gratia *et al*., where a quantity of Cs higher than 3% permits to circumvent the crystallization via environmentally sensitive and defect-predisposed intermediate hexagonal phases^36^. Furthermore, the presence of PbI_2_ (diffraction peak located at 2*θ* ≈ 12.6°) is clearly identifiable for the 0% Cs compound and can be associated with the excess of lead and halogen species present in the precursor solution compared to the total monovalent cation concentration. This diffraction peak disappears when enough Cs is introduced in the precursor solution for fresh devices. On the other hand, it is worth noting that even for the highest Cs content, no specific peak of CsI were observed. Cross-section SEM image (taken after focused ion beam polishing, FIB) of a complete opaque PSC is presented in Fig. S3. It is composed of a perovskite absorber layer grown with 5% Cs, using FTO as TCO, a compact TiO_2_ and a mesoporous TiO_2_ as electron selective contact, Spiro-OMeTAD as hole selective contact and Au as rear cathode. The best efficiency has been obtained with a submicrometre perovskite film that ensures a proper light harvesting together with an effective charge collection. This SEM image confirms the very compact structure of the perovskite absorber which is composed of large grains. A summary of *J-V* characteristics of opaque cells for the different absorber formulations is presented in Fig. S4. As expected, the addition of a small amount of caesium (5% Cs) in the precursor solution conducting to an overall improvement of the cell performances as reported in literature^37^, since short circuit current (*J*_*SC*_) is improved almost 2 mA cm^−2^, with a moderate increment of fill factor (FF), keeping circuit voltage (*V*_*OC*_) and with a reduction of hysteresis phenomena compared to Cs-free perovskite. Therefore, average PCE was increased significantly with a champion cell reaching PCE = 19.7% with a steady-state PCE = 19.5% (Fig. S5). However, in literature a 10% Cs concentration led to results very near to the record ones obtained at 5% Cs^37^, while, in our case, the introduction of 10% Cs resulted excessive, reducing very significantly the *J*_*SC*_ and FF regarding 5% Cs concentration and not presenting PCE enhancement in comparison with Cs-free perovskite absorber.

### Optimization of the ITO deposition process

In most of the reported fabrication procedures for semitransparent PSC, a thin protection layer, typically molybdenum oxide^9^ is deposited on the Spiro-OMeTAD prior to the deposition of transparent conductive oxide in order to protect the organic material during the sputtering process. To reduce the number of steps during cell fabrication process, a procedure buffer layer-free with a direct growth of indium tin oxide (ITO) layer on the top of the organic *p*-type extraction layer was explored in this work. For this purpose, the sputtering deposition had to be carefully controlled to avoid damages of the Spiro-OMeTAD layer.

**Figure 1:**
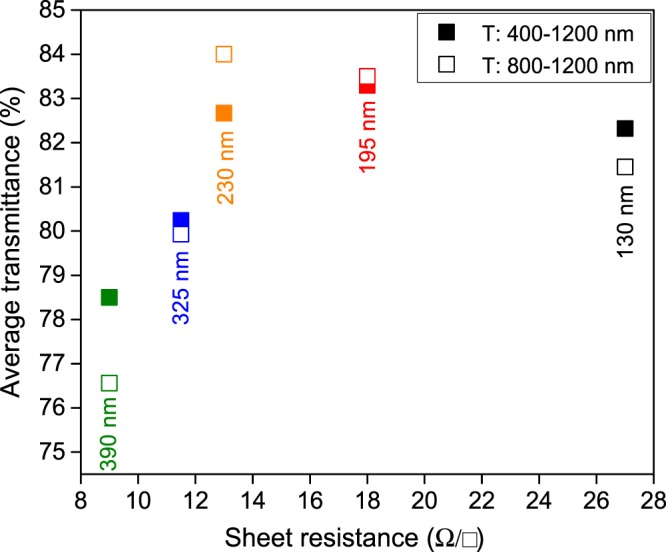
Average transmittance of ITO layers deposited onto borosilicate glass substrate by RF magnetron sputtering over the 400–1200 nm (hollow squares) and 800–1200 nm (full squares) ranges, as function of thickness and sheet resistance.

To obtain ITO films with both high NIR transparency and conductivity, several deposition parameters such as pressure, RF power and deposition time were varied while films were deposited at room temperature onto borosilicate glass substrates. Thus, thickness was varied from 130 nm to 390 nm to study its effect on the optoelectronic properties of the contact. Then, the procedure described in the experimental part leads to ITO films with mobility and carrier concentrations of 46 cm^2^ V s^−1^ and 3.6 × 10^20^ cm^−3^ respectively. This values were not essentially affected by the layer thickness that mostly impacts on the transparency of the film. In Fig. 1 the average transmittance for the full spectrum (*λ* = 400–1200 nm) and its near infrared part (*λ* = 800–1200 nm) *vs* sheet resistance of ITO layers with different thicknesses are shown. Full transmission spectra are given in supplementary materials (Fig. S6). An average transparency near 83% was obtained in the 400–1200 nm region for 195 nm and 230 nm thick films, and this value resulted even higher for the NIR part (84%) for the 230 nm thick ITO despite the high doping level of the material. By a further decrease of the layer thickness, not only an augmentation of overall transmittance was not perceived due to the apparition of interferences but also the expected increase of the sheet resistance was observed. Thicker films, *i.e*. 325 and 390 nm resulted poorly transparent. In summary, a suitable balance between the transparency and the sheet resistance is obtained for the 230 nm thick ITO thin film presenting *R*_*sheet*_ = ~13 Ω/square and transmittance over 80% for the full spectra with average *T* = 84% for the 800–1200 nm region.

### Fabrication and characterization of Semitransparent PSC

**Figure 2:**
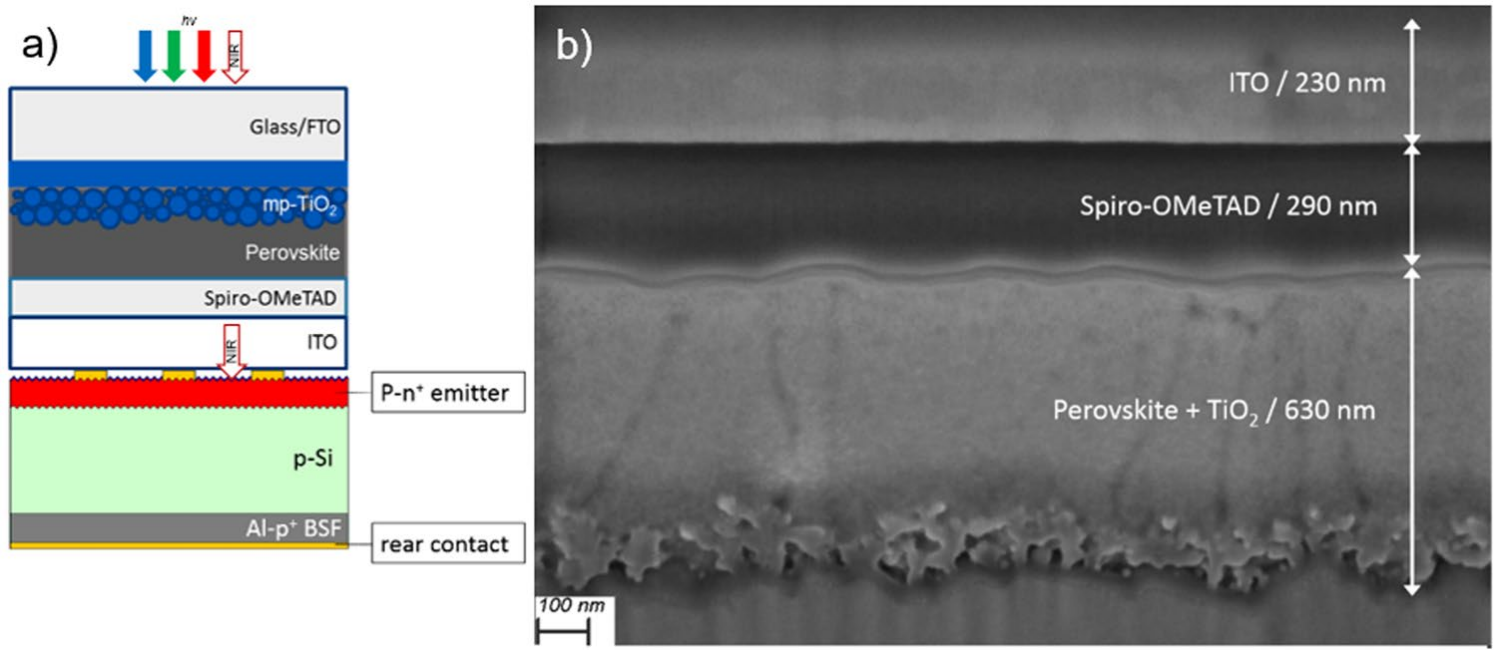
(**a**) Scheme of the 4-terminal tandem device proposed in this work, with a semitransparent PSC MoO_x_-free with ITO rear contact as top cell and Al-BSF Si solar cell as bottom one to absorb the near infrared (NIR) part of the spectra. (**b**) Cross-sectional Scanning Electron Microscopy image of a complete semitransparent PSC MoO_x_-free terminated with a 230 nm thick ITO contact, made by Focused Ion Beam polishing and Scanning Electron Microscopy imaging.

**Figure 3:**
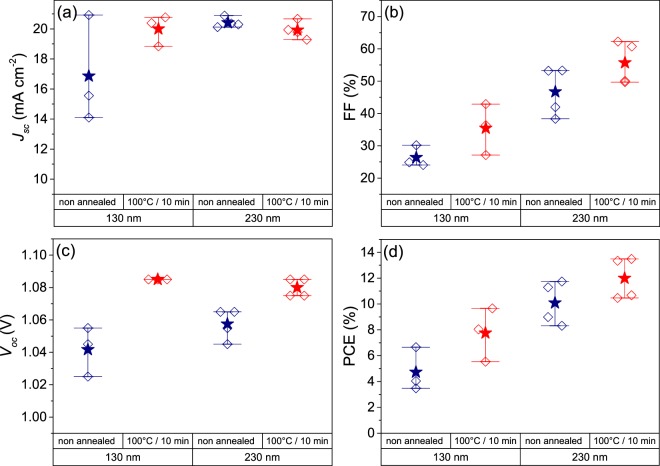
Photovoltaic parameters as the function of the ITO thickness before (blue)/after (red) annealing on small semitransparent PSC previously the Au grid deposition. (**a**) Short-circuit current density (*J*_*SC*_), (**b**) fill factor (FF), (**c**) open-circuit voltage (*V*_*OC*_) and (**d**) power conversion efficiency (PCE). Mean values are represented as stars and whiskers represent the interval of maximum-minimum values attained.

**Figure 4:**
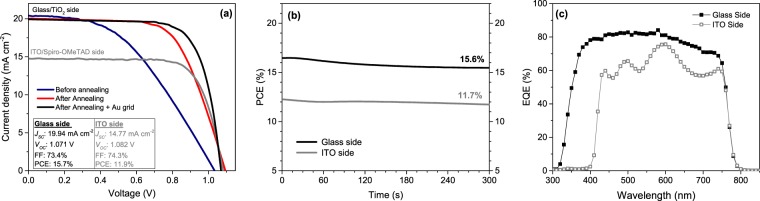
(**a**) *J-V* characteristics for semitransparent PSC with small size (0.16 cm^2^ active area) containing 230 nm thick ITO. The illumination was carried out both from typical *n*-type side (glass/FTO/bl-TiO_2_/mp-TiO_2_) before annealing (blue), after annealing (red) and after annealing with evaporated Au grid (black) and through the *p*-type one (ITO/ Spiro-OMeTAD) after annealing and Au grid evaporation (grey); scan rate 80 mV s^−1^. (**b**) Steady-state efficiencies at *V*_*MPP*_ for the fabricated semitransparent PSC with small size with illumination through glass side (black) and through ITO one (grey). (**c**) External quantum efficiency for illumination through the *n*-type side (glass side): full black squares and through the *p*-type side (ITO side): hollow grey squares.

The first step to obtain a 4 T tandem device with the architecture presented in Fig. 2a was the preparation of a small size (0.16 cm^2^ active area) semitransparent PSC. According previous results, two different ITO thicknesses (130 and 230 nm) were tested as rear transparent contact for these perovskite solar cells in substitution of metallic Au cathode. Although 230 nm thick samples showed *a priori* the most adequate opto-electronical properties since good transmittance in NIR and low resistivity, 130 nm thick samples were also tested at the same time to reduce the sputtering time of ITO in order to limit or prevent possible damages. The morphology of this device is shown in cross sectional FIB-SEM figure (Fig. 2b) for a complete semitransparent PSC containing 230 nm thick ITO as rear contact, observing similar morphology and thickness for the different layers employed regarding the opaque PSC previously detailed, with a dense ITO film, well-attached to Spiro-OMeTAD and without revealing any remarkable degradation or damaging driven by sputtering deposition of ITO rear contact. In addition, the annealing of ITO resulted an important parameter to keep under control. Thus, photovoltaic parameters as a function of ITO thickness and with/without annealing at 100 °C are summarized in Fig. 3. Solar cells were systematically built for each condition and the illumination of the cell was carried out from the glass side through FTO. Before annealing, PSC prepared with 130 nm thick ITO layer did not show performances superior to 6.8%, with *J*_*SC*_ that drastically vary from one cell to another and FF not exceeding 30%. Performances are strongly improved with a thicker ITO film (230 nm), detecting samples with *J*_*SC*_ higher than 20 mA cm^−2^ as a result of an excellent transmittance of ITO layer, with a coherent enhancement of FF and *V*_*OC*_ principally related to the reduction of ITO sheet resistance for a final maximum PCE of 11.8%. However, a thermal post treatment was found necessary to further increase the device performances by reducing series resistance issues (from 24.40 to 12.64 Ω cm^2^), exhibiting a great impact mainly on FF independently the ITO thickness, leading to a maximum PCE of 14.2% for the semitransparent PSC with 230 nm thick ITO. This reduction of series resistance on annealed samples can be associated to a better lateral collection and presumably to a better connection between Spiro-OMeTAD and ITO. The evolution of *V*_*OC*_ can be related to the reduction of the recombination losses. As the Spiro-OMeTAD/ITO interface is the most temperature sensitive part of the photovoltaic device, the decrease of trap density located at this interface and acting as non-radiative recombination centres could explain the improvement of this parameter by a better intimate contact of both materials as well. Anyway, as the fill factor still remains the limiting parameter due to high series resistance, a gold metallic grid was thermally evaporated onto ITO in order to enhance the lateral collection of the photogenerated carriers (see Fig. S7). As shown in Fig. 4, the collection grid further diminished series resistance (up to 5.79 Ω cm^2^) and permitted to attain a FF of 73.4% for an overall efficiency of 15.7% (15.6% in steady-state). In order to check the semitransparency of the fabricated PSC, it was also illuminated through ITO side, lowering importantly the *J*_*SC*_ to 14.77 mA cm^−2^ due to parasitic absorptions typical in Spiro-OMeTAD doped with cobalt complexes salts in the ranges of wavelengths 410–560 nm and 625–750 nm as expected from literature for Co-doped Spiro-OMeTAD^38^. This photocurrent is not far from the best values reported with rear illumination of semitransparent PSC^9^. In this case, FF and *V*_*OC*_ are not very significantly affected for a final PCE = 11.9% (11.7% in steady-state conditions), with illumination through ITO side.

**Figure 5:**
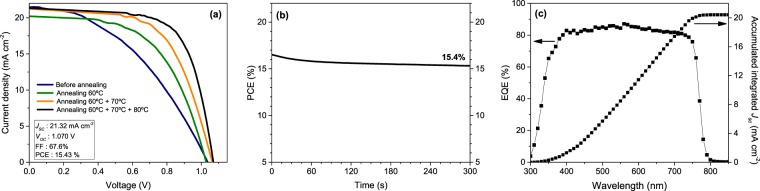
(**a**) *J-V* characteristics for best-performing semitransparent PSC with big size (0.64 cm^2^ active area) with 230 nm thick ITO rear contact and containing a gold metallic grid as function of the annealing temperature (before annealing: blue, annealed at 60 °C: green, annealed at 60 °C + 70 °C: orange and annealed at 60 °C + 70 °C + 80 °C: black); scan rate 80 mV s^−1^. (**b**) Steady-state PCE measured at *V*_*MPP*_ for the semitransparent PSC with larger size using front illumination. (**c**) External quantum efficiency and accumulated integrated short-circuit current density for semitransparent PSC with big size.

After proving the efficient fabrication of MoO_x_-free semitransparent PSC with sputtered ITO as rear contact in small size, larger devices of 0.64 cm^2^ active area were made in a similar manner, choosing 230 nm thick ITO for the experiments. However, heating protocols were found not directly transferable on a larger device, since annealing at 100 °C during 10 min dramatically reduced the efficiency in larger PSC. Consequently, thermal treatment conditions were adjusted to circumvent undesirable damaging of samples and so avoiding reduction of PCE. Thus, in the case of 0.64 cm^2^ semitransparent PSCs, consecutive annealing steps had to be carried out at 60, 70 and 80 °C during 5 minutes to improve the performance of the samples. The evolution of the *J-V* curve and main electrical parameters as a function of the applied temperature are presented in Fig. 5 and Fig. S8 respectively (devices terminated with a gold collection grid). As observed before, the annealing post-treatment mainly pushed up FF (from 41.9% to 67.6%) by reducing the series resistance *R*_*s*_ (from 20.55 Ω cm^2^ before annealing until 7.14 Ω cm^2^ with the whole sequential heating protocol here described) and also *V*_*oc*_ in lesser manner (from 1.037 V to 1.070 V) with an exceptionally high *J*_*SC*_ (~21 mA cm^−2^). Furthermore, *J*_*SC*_ obtained from EQE integration in non-dynamic conditions (20.42 mA cm^−2^) showed minor differences with the *J*_*SC*_ from *J-V* characterization. Therefore, a record 15.43% device (15.4% at steady-state) was obtained by a careful control of deposition and heating conditions in MoO_x_-free ITO-rear-contacted semitransparent PSC with larger area.

### Bifacial characterization of Semitransparent PSC by time-resolved fluorescence imaging

Charge carrier transport and recombination processes in the semitransparent PSC were studied by time-resolved photoluminescence (TRPL). Using our time-resolved fluorescence imaging set-up, various orders of magnitude for the incoming photon flux were used, and so the bulk charge carrier concentration during the decay was tuned between 10^13^ cm^−3^ and 2 × 10^16^ cm^−3^. Resulting transient signal was fitted using a model including shallow traps, interface recombination and in-depth diffusion of charge carriers, remaining valid for the interval of charge carrier concentrations here considered. Consequently, diffusion lengths and lifetimes at solar concentration were successfully determined using the equations derived in the supporting information.

**Figure 6:**
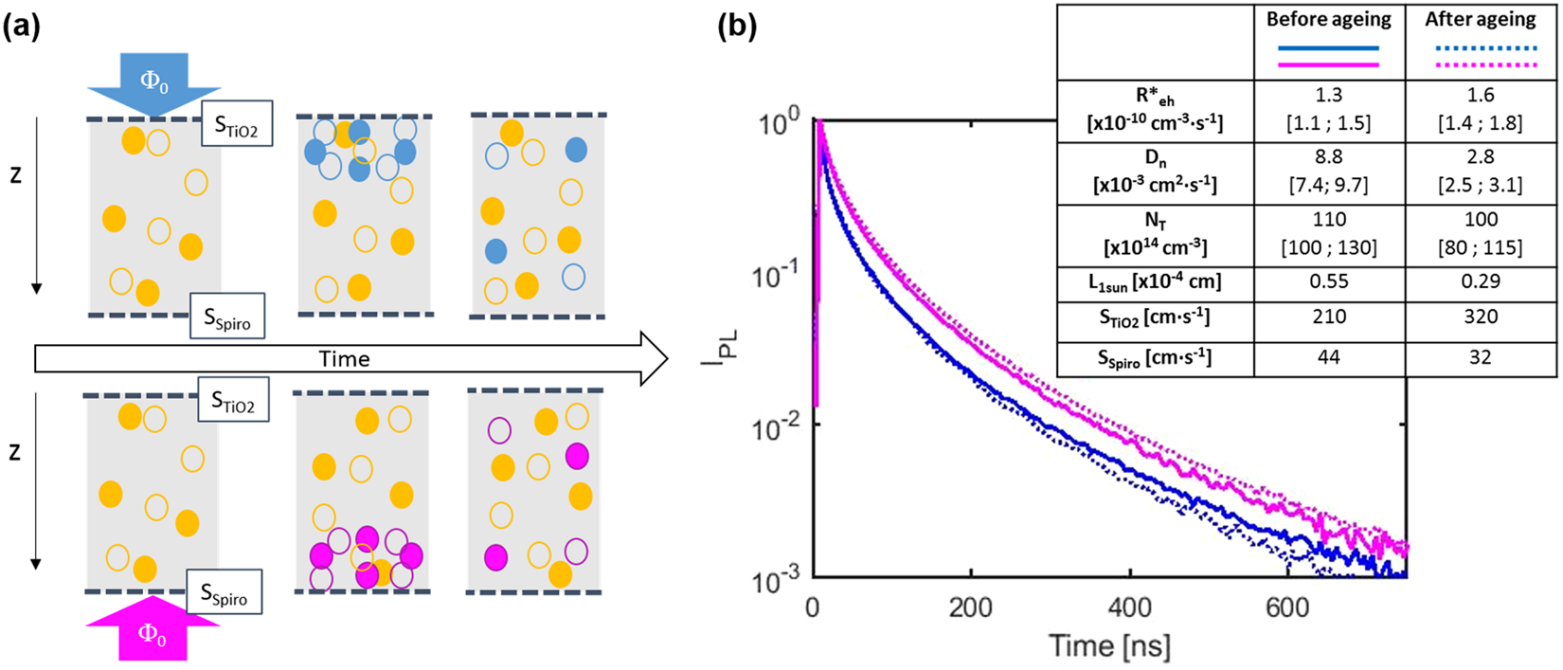
(**a**) Schematic representation of the TRPL measurement. Full circles stand for electrons, while empty ones for holes. Blue symbols stand for photo-generated charge carriers at TiO_2_ side, pink ones at Spiro-OMeTAD side, whereas yellow ones stand for trapped charges. (**b**) TRPL transients acquired at incident photon flux *Φ*_*0*_ = 1.2 × 10^12^ pulse^−1^ cm^−2^, illuminating on both TiO_2_ (blue) and Spiro-OMeTAD (purple) sides, before (full lines) and after (dotted lines) 20 days ageing. Inset table displays fitted values and confidence intervals extracted for the external radiative recombination rate R*_eh_, the diffusion coefficient *D*_*n*_, the shallow trap concentration *N*_*T*_ and the interface recombination velocities *S*_*TiO2*_ and *S*_*Spiro*_. The diffusion length *L*_*1sun*_ is calculated under steady state illumination (1 sun), as explained in the SI.

By an optical excitation (LASER pulse) on both sides of the record big semitransparent PSC (glass/FTO/bl-TiO_2_/mp-TiO_2_ abbreviated as TiO_2_ side, and ITO/Spiro-OMeTAD coded as Spiro-OMeTAD one), the differences on TRPL response between both illumination sides were investigated. This process is sketched in Fig. 6, where experimental photoluminescence (PL) transients acquired at photon flux *Φ*_*0*_ = 1.2 × 10^12^ pulse^−1^ cm^−2^ are shown for a fresh device (solid lines) and after 20 days ageing (dotted ones). Decays at TiO_2_ side are coloured in blue while pink was employed for Spiro-OMeTAD side. For all cases, PL decay was accelerated at shorter times after the optical pulse, when charge carriers recombine faster. This is due to their higher concentration next to the interface, which can be explained through the combination of a high absorption coefficient (*α*_*532*_ = 10^5^ cm^−1^)^39,40^, with a slow in-depth diffusion in bulk perovskite. No significant changes were detected with ageing for semitransparent PSC samples when they were illuminated through Spiro-OMeTAD side, which indicates not only a relatively quite stable Spiro-OMeTAD/perovskite interface but also a damageless ITO sputtering process since the behaviour of the cell in terms of generation and collection of charges is not limited or reduced when Spiro-OMeTAD side illumination was applied. In contrast, the TRPL decay is slightly accelerated after ageing for TiO_2_ side illumination. This points out a better quality and stability of Spiro-OMeTAD/perovskite interface than perovskite/TiO_2_ one despite the potentially damaging ITO sputtering process. In addition, the stability of PSC has been reported to be limited by TiO_2_ interface due to ultraviolet light-induced instability of titania^41,42^. In order to quantitatively compare the diffusion and recombination processes in the fresh and aged device, a kinetics model combining trap-assisted recombination and in-depth diffusion of charge carriers has been developed (briefly described in supplementary information). It leads to an accurate reconstruction of experimental transients acquired for different orders of magnitude of *Φ*_*0*_ using both illumination directions, providing a precise estimation of the physical properties linked to recombination and diffusion processes. More precisely, the weight of different recombination pathways by determining the external radiative recombination rate *R*_*eh*_ and the shallow trap concentration *N*_*T*_ was assessed. Also, the diffusion coefficient *D*_*n*_ for holes and electrons and the recombination velocity associated to ETL/perovskite and HTL/perovskite interfaces was evaluated. As stated before, these parameters were independent from the injection level considered, on the contrary to the lifetimes frequently determined with a bi-exponential model^41,43^. Parameters resulting from the fitting procedure are displayed in the inset table in Fig. 6, along with their confidence intervals. Consistently with the fact that the illumination on the TiO_2_ side led to faster PL decay at short times, a higher concentration in recombination sites at the interface formed with TiO_2_ was obtained. This can be explained by a poor intimate contact between TiO_2_ and perovskite, but also by the significant larger surface area of the mesostructured TiO_2_, so reducing the electronic quality of the interface. In any case, the effective amount of recombination at this interface resulted higher than at the Spiro-OMeTAD/perovskite one. Concerning the effect of ageing, fitted values for S_TiO2_ and S_Spiro_ confirmed the abovementioned analysis on the better stability of Spiro-OMeTAD/perovskite interface in comparison with perovskite/TiO_2_ one. Although the observed faster decay could be related with a better injection towards the charge transport layers, it doesn’t seem to play a predominant role in this case since this injection generally takes place at the ps scale^35^ and is frequently observed at short circuit conditions^44^, which doesn’t apply here. Regarding bulk perovskite, estimated external radiative recombination coefficient values were close to previously reported ones, indicating the negligible impact of deep defects on charge carrier transport^45–47^. However, the shallow trap concentration valuates around 10^16^ cm^−3^, which is remarkably higher than the one measured for a half-cell (*N*_*T*_ = 4.2 × 10^14^ cm^−3^ on FTO/bl-TiO_2_/mp-TiO_2_/perovskite) with the same chemical composition for the absorber^48^. This might be a consequence of the deposition of the Spiro-OMeTAD/ITO structure upon the perovskite. Therefore, diffusion coefficient *D*_*n*_ by simply using Einstein relation (*D* = *μkT*/*q*), provided a mobility of *μ*_*n*_ = 0.35 cm^2^ V^−1^ s^−1^, inside the range of the reported values for polycrystalline solution-processed perovskites but relatively distant from the record values^45–47^. Furthermore, charge collection remains being effective as previously determined recombination parameters correspond to a lifetime of *τ* = 340 ns at 1-sun illumination. Generally speaking, ageing process mainly reduced the diffusion coefficient (*D*_*n*_), consequently reducing *J*_*sc*_ by collection issues. On the other hand, radiative and trap-assisted recombination pathways resulted less affected by ageing, which is consistent with *V*_*OC*_ displayed in the evolution of photovoltaic parameter in Fig. S9. In conclusion, perovskite/TiO_2_ interface resulted more limiting and sensitive to ageing than Spiro-OMeTAD/perovskite one for the semitransparent device characterized by bifacial TRPL, validating our MoO_x_-free ITO deposition process by magnetron sputtering as a damageless and non-harmful technique to deposit rear transparent contacts in semitransparent PSC.

### 4-T Tandem solar cell assembly

Electroluminescence (EL) measurement of a Si solar cell (10 × 10 mm) extracted from a standard and commercially-available Al-BSF silicon wafer (156 × 156 mm) by laser-cutting is shown in Fig. S10. The lower response on the complete left, bottom and right edges, likely originates from some damages of the emitter due to the heat-affected zone after laser cut. The upper edge does not show this degradation because the cut was performed a few mm away behind the bus bar. The EL is also affected in the upper right corner due to the presence of the Ag pad behind the bus bar which was used for soldering the measurement contact. While the Al-BSF silicon wafer showed a 19.52% of PCE, the emergence of recombination losses in those areas causes an overall *V*_*OC*_ reduction of 20–40 mV, decreasing the efficiency of the reduced-size Si cell to 18.53%.

**Figure 7:**
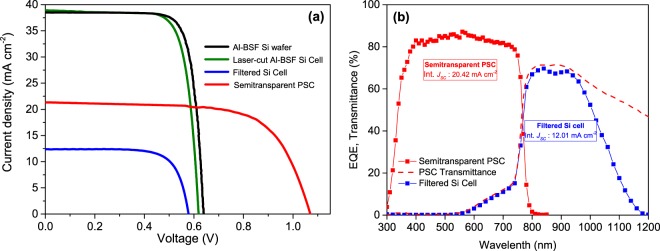
Performances of the 4-T Tandem perovskite/Al-BSF Silicon. (**a**) *J-V* characteristics for the commercially-available Al-BSF Si wafer as received (black), laser-cut Al-BSF Si Cell (green), filtered laser-cut Al-BSF Si Cell (blue) and champion semitransparent PSC with 230 nm ITO rear contact with big area (red). (**b**) External Quantum Efficiency (EQE) measurements of the semitransparent PSC employed as top cell (red line with red squares) and the filtered silicon solar cell used as bottom one (blue line with blue squares). Dashed red line corresponds to transmittance spectra of semitransparent PSC.

In Fig. 7 *J-V* curves and EQE measurements for silicon devices and champion semitransparent PSC with big size are included. After filtering, Si solar cell showed a *J*_*SC*_ of 12.75 mA cm^−^² using an index matching fluid to reduce the optical losses due to the air interfaces, with a *V*_*OC*_ = 0.585 V and FF = 77.0% for a final overall PCE of 5.75%. In Fig. 7b, the transmittance of the semitransparent PSC is also included reaching a maximum transparency of 72% at 850 nm. To the best of our knowledge the highest transmissions at this wavelength published so far are 80%^22^ and 84%^14^ but these results were obtained using antireflection coatings, not implemented here. For example, Duong *et al*. deposited a MgF_2_ on the rear side and a textured foil was used on the front side^14^. When the transmittance spectra of top semitransparent PSC is compared with the EQE of filtered Si, insignificant losses from non-absorbed photons are observed, since for wavelengths <950 nm, practically all transmitted photons from perovskite device are collected by silicon, whereas for wavelengths >950 nm an important part of transmitted photons from semitransparent PSC are not collected in filtered silicon cell due to reflection losses in original Al-BSF Si wafer (Fig. S11). In Table 1 photovoltaic parameters of the Si solar cell on bottom and champion semitransparent PSC with big size are summarized. Finally, adding the efficiencies obtained for that champion semitransparent PSC to the filtered silicon cell, a maximum efficiency of 21.18% can be claimed for a 4 T tandem device, corresponding to a 1.66% absolute PCE boosting regarding the standard commercially available Al-BSF wafer and also a 2.65% over the silicon cell after being cut.

**Table 1:** Photovoltaic performances of the devices used for 4-T semitransparent PSC/Al-BSF Si assembly.

Device	*J*_*SC*_ (mA cm^−^²)	*V*_*OC*_ (V)	FF (%)	PCE (%)
Opaque PSC	22.56	1.116	78.2	19.70
Semitransparent PSC	21.32	1.070	67.6	**15.43**
Al-BSF Si wafer	38.46	0.639	79.4	19.52
Laser-cut Al-BSF Si Cell	38.86	0.619	77.0	18.53
Filtered Si Cell	12.75	0.585	77.0	**5.75**
4 T Tandem Perovskite/Si	—	—	—	**21.18**

Electrical parameters (*J*_*SC*_, *V*_*OC*_, FF and PCE) extracted from *J-V* curves measured at 80 mV s^−1^ for the different champion solar cells.

## Conclusions

In this work we have described the fabrication of highly efficient MoOx-free semitransparent perovskite solar cells and their use as top cell of a 4-T tandem devices. With the optimization of perovskite composition and ITO properties (thickness, transmission, sheet resistivity and annealing time), record efficiencies up to 15.7% on a 0.16 cm^2^ and 15.43% on 0.64 cm^2^ have been achieved with semitransparent perovskite cell containing rear ITO cathodes deposited by RF magnetron sputtering without any MoO_x_ intermediate film between Spiro-OMeTAD and ITO. Up to our knowledge, it constitutes the record efficiency for buffer layer-free semitransparent PSCs. Moreover, the tandem device built in combination with a commercially-available Al-BSF silicon leads to a substantial enhancement of the original silicon performance: up to 1.66% in absolute PCE regarding the wafer, even 2.65% when the reduced Si solar cell size is taken into account, since 21.18% efficiency has been obtained for the abovementioned perovskite/silicon 4-T tandem device. This result can be further improved by working on the infrared transmission in the PSC as well as minimizing the losses related to the Si wafer cutting. Additionally, the main physical properties linked to recombination and diffusion processes of the semitransparent cell have been evaluated from time-resolved-photoluminescence (TRPL) imaging measurement carried out on both sides of the semitransparent cell. This characterization shows that Perovskite/TiO_2_ interface was generally the most critical part of the final semitransparent device from a recombination point of view, revealing that the introduction of a transparent high-quality sputtered-ITO as cathode onto Spiro-OMeTAD without any buffer layer in between does not constitute a bottleneck for semitransparent PSC fabrication.

## Methods

### Perovskite Solar Cell Fabrication

FTO-coated glass substrates (TEC 10, 3 mm thick) purchased from Ossila were chemically etched using Zn powder and HCl (4 M). Then, the substrates were ultrasonicated (60 min in 2% RBS detergent solution in deionized water, 25 min in isopropanol), rinsed with isopropanol and heated at 500 °C to remove any organic residue. A TiO_2_ compact layer was deposited by spray pyrolysis at 450 °C using O_2_ as carrier gas and a precursor solution made of titanium diisopropoxide bis(acetylacetonate) (0.6 mL, 75% wt. in isopropanol, Sigma Aldrich), acetylacetone (0.4 mL, Sigma Aldrich) in absolute ethanol (9 mL, Sigma Aldrich). A mesoporous TiO_2_ layer *ca*. 150 nm was deposited by spin-coating employing a solution of 30NR-D (Dyesol) in absolute ethanol (1:7 in weight) and subsequently heated to sinter the TiO_2_ particles (5 min at 125 °C, 5 min at 325 °C, 5 min at 375 °C, 15 min at 450 °C and 30 min at 500 °C). During cooling down, samples were introduced into a N_2_ glove box to continue the fabrication. A (MA_0.166_FA_0.833_)Pb(Br_0.166_I_0.833_)_3_ perovskite solution, also referred 0% Cs or (MA,FA)Pb(Br,I)_3_, was prepared by dissolving 1.10 M PbI_2_ (TCI Chemicals), 0.20 M PbBr_2_ (Alfa Aesar), 1.00 M formamidinium iodide (FAI, Dyesol) and 0.20 M methyl ammonium bromide (MABr, Dyesol) in a mixture of DMSO:DMF (4:1 in v/v) as solvent. To obtain the final triple cation perovskite, *i.e*. Cs_x_(MA_0.166_FA_0.833_)_1−x_Pb(Br_0.166_I_0.833_)_3_, the required quantity of Cs^+^ was added from of a stock precursor solution of CsI (Sigma Aldrich) 1.50 M in DMSO according to Saliba *et al*.^37^. Perovskite solutions were deposited by spin coating with minor modifications regarding Saliba *et al*. work^37^, using a double *plateau* (2000 rpm to cast the perovskite precursor solution followed by 6000 rpm to drip 100 µL of chlorobenzene). Then, samples were annealed at 100 °C during 30 min. A solution of Spiro-OMeTAD (2,2′,7,7′-tetrakis(*N,N*-di-p-methoxyphenyamine)−9,9-spirobifluorene, Merck), 110 mg mL^−1^ in chlorobenzene (Sigma Aldrich, anhydrous) including tris(2-(1H-pyrazol-1-yl)-4-*tert*-butylpyrydine)cobalt(III) bis(trifluoromethylsulphonyl)imide (5 mol% regarding Spiro-OMeTAD, FK209, from Dyesol), lithium bis(trifluoromethylsulphonyl)imide (50 mol% regarding Spiro-OMeTAD, LiTFSI, from Sigma Aldrich) and 4-*tert*-butylpyridine (330 mol% regarding Spiro-OMeTAD, *t*-BP, from Sigma Aldrich 96%wt. purity) was spin coated at 3000 rpm for 20–25 s.

In the case of opaque cells, 100 nm of Au were thermally evaporated under high vacuum as cathode. For semitransparent devices, ITO was deposited according the following section. Over ITO cathodes, a U-shape pattern of Au (~100 nm thick) was thermally evaporated over the borders of ITO area in order to minimize the series resistance.

### Rear contact deposition process

The ITO deposition system consists in 3 magnetrons of 3-inch diameter targets mounted in a high vacuum chamber. In this system, the targets are positioned in confocal geometry over the substrate, the inter-electrode distance is approximately 10 cm in balanced magnetron mode and substrates are mounted on a stainless steel rotating disk. The indium oxide and tin oxide fluxes are generated by RF (13.56 MHz) magnetron sputtering from an ITO (90% In_2_O_3_ − 10% SnO_2_) target. It is important to remark that for this process only one target was used and RF system was chosen to reduce physical impact in comparison with direct-current sputtering.

Prior all depositions, the sputtering chamber was evacuated to P < 5 × 10^−7^ mbar and the ITO target pre-sputtered for at least 10 minutes in Ar plasma at 2.5 × 10^−3^ mbar to remove the surface pollution. For the analysis of transmission spectra of ITO films onto glass, 2 mm thick borosilicate glass was used as substrate. When ITO films were applied to semitransparent PSC, both glass substrates (for control purposes) and semitransparent perovskite devices were introduced in the deposition reactor, where the working pressure and RF power were fixed to 2.5 × 10^−3^ mbar and 50 W respectively. ITO annealing was made at 100 °C during 10 minutes for small semitransparent PSCs; whereas, for larger devices, the annealing procedure was design with consecutive heating steps at 60, 70 and 80 °C for 5 min onto a hot plate separated by a cooling down to room temperature in air in between heating steps.

### Photovoltaic Characterization

In order to analyse the photovoltaic properties of the solar cells, an AAA sun simulator (Oriel Sol3A) was employed as light source using an AM1.5 G spectra and 1 Sun (100 mW cm^−2^) intensity. A digital source meter (Keithley Model 2400) was used to apply a voltage to the cell while photocurrent was registered in order to record *J*-*V* curves. The voltage sweeping rate was fixed to 80 mV s^−1^ unless other one was specified. For steady-state output performance, PCE was plotted as function of time at maximum power point voltage (*V*_*MPP*_) conditions under continuous illumination using the same light source employed for *J-V* measurements (Oriel Sol3A sun simulator with AM1.5 G spectra and 1 sun intensity). For quantum efficiency measurements, an Oriel IQE200 system in connection with a source meter (Keithley 2400) were utilized to record the performance using a digital lock-in amplifier (Oriel Merlin). Both opaque cells and small semitransparent ones presented an active area of 0.16 cm^2^, using a black metal laser-cut mask to illuminate 0.09 cm^2^. Larger semitransparent PSC were fabricated with an active area of 0.64 cm^2^ being illuminated 0.25 cm^2^ with a black metal laser-cut mask as well.

### 4-T tandem assembly

To fabricate a 4 T tandem solar cell, a standard (156 × 156 mm) Al-BSF silicon wafer, supplied by the cell and module producer Photowatt, with PCE = 19.52% (*V*_*oc*_ = 639.2 mV, *J*_*sc*_ = 38.46 mA cm^−^² and FF = 79.4%) was used for bottom cell. The cell was down-sized by creating shallow patterned lines at the cell back side with a Nd:YAG laser operated at 10 kHz with a 1064 nm wavelength. The weakened lines were then cleaved so as to create smaller pieces with 1 cm² active area (including 6 metallic fingers). Then, two electrical wires were soldered on the front and back of the reduced Si cell to keep bottom cell contacts when top cell was stack over. In order to evaluate the performances of the perovskite/silicon 4 T tandem device, 2 × 2 cm blank perovskite devices were fabricated to be used as top filters for the 1 cm^2^ Si Al-BSF bottom solar cell, enabling to measure the whole Si area. An index matching material was placed between filter and Si bottom cell. Those blank perovskite devices used as filters consisted in real functional semitransparent PSC, optically identical to the ones reported in this work, with the only absence of the U-shape Au contact evaporated on the surroundings of the ITO area designed to reduce the series resistance. Perovskite filters of larger size than Si bottom cell were intentionally used to ensure the absence of unfiltered light incident onto Si bottom cell that would artificially increase the photocurrent. Moreover, blank perovskite devices were fabricated exactly under identical conditions than the here reported semitransparent PSC to ensure the reliability of the method: during the same day, in the same batch and employing the same solutions and deposition conditions. Consequently, the overall performance of 4-T perovskite/Silicon tandem solar cell was calculated by adding the efficiencies of the semi-transparent PSC and the filtered silicon bottom one. In order to accurately determine the EQE of bottom cell, the Si cell was illuminated through our champion semitransparent PSC with 0.64 cm^2^ active area.

### TRPL Imaging

The main components of the Time-Resolved Photoluminescence (TRPL) setup are a pulsed laser (TALISKER, Coherent) delivering 15 ps wide pulses (*λ* = 532 nm, repetition rate *f*_0_ = 40 kHz) and triggering an intensified electron-multiplied CCD (em-ICCD) time-gated camera (PIMAX4, Princeton instruments). The camera gating width was fixed at 2 ns, while its gating time was varied stepwise from *t* = *t*_*pulse*_ (taken as reference) and *t* = 1 μs. The wide-field illumination is obtained due to a home-built opto-mechanical setup that filters out laser speckles. The intensity of the incident photon flux was varied over three order of magnitude (*Φ*_*0,1*_ = 2.5 × 10^10^; *Φ*_*0,2*_ = 2.25 × 10^11^; *Φ*_*0,3*_ = 1.6 × 10^12^ pulse^−1^ cm^−2^). The illuminated area (≈5.2 mm^2^) significantly overlaps the collection area (≈1 mm^2^). The typical diffusion area for a charge carrier in the sample (≈1–10 μm^2^) is itself significantly smaller than the collection area. Hence, lateral diffusion of charge carriers does not lead to any artefacts^49^.

## Electronic supplementary material

Supplementary Information

## Data Availability

The data that support the findings of this published article (and its Supplementary Information files) are available from the corresponding author on reasonable request.
