# Supplementary material for: Highly efficient MoOx-free semitransparent perovskite cell for 4 T tandem application improving the efficiency of commercially-available Al-BSF silicon
Source: Sci Rep. 2018 Oct 31;8:16139. doi: 10.1038/s41598-018-34432-5 (PMC6208347; doi:10.1038/s41598-018-34432-5)
Supplement: Supplementary file 1 — Supplementary Information [file 41598_2018_34432_MOESM1_ESM.pdf]

Highly efficient MoO_x-free semitransparent perovskite cell for 4T tandem application improving the efficiency of commercially-available Al-BSF silicon

Supporting Information

F. Javier Ramos[1,2]*, *Sebastien Jutteau*[1,3], *Jorge Posada*[1,3], *Adrien Bercegol*[1,3], *Amelle Rebai*[1], *Thomas Guillemot*[4], *Romain Bodeux*[1,3], *Nathanaelle Schneider*[1,2], *Nicolas Loones*[1,3], *Daniel Ory*[1,3], *Cedric Broussillou*[5], *Gilles Goaer*[5], *Laurent Lombez*[1,2], *Jean Rousset*[1,3]*

[1] IPVF, Ile-de-France Photovoltaic Institute (IPVF), 30 Route Départementale 128, 91120 Palaiseau, France
[2] CNRS, Ile-de-France Photovoltaic Institute (IPVF), UMR 9006, 30 route départementale 128, 91120, Palaiseau, France
[3] EDF R&D, 30 Route Départementale 128, 91120 Palaiseau, France
[4] Licorne Laboratory, ECE Paris, 37 quai de Grenelle, 75015 Paris, France
[5] Photowatt, EDF ENR PWT, 33 rue Saint-Honoré, Z.I. Champfleuri, 38300 Bourgoin-Jallieu, France

*javier.ramos@cnrs.fr, jean.rousset@edf.fr

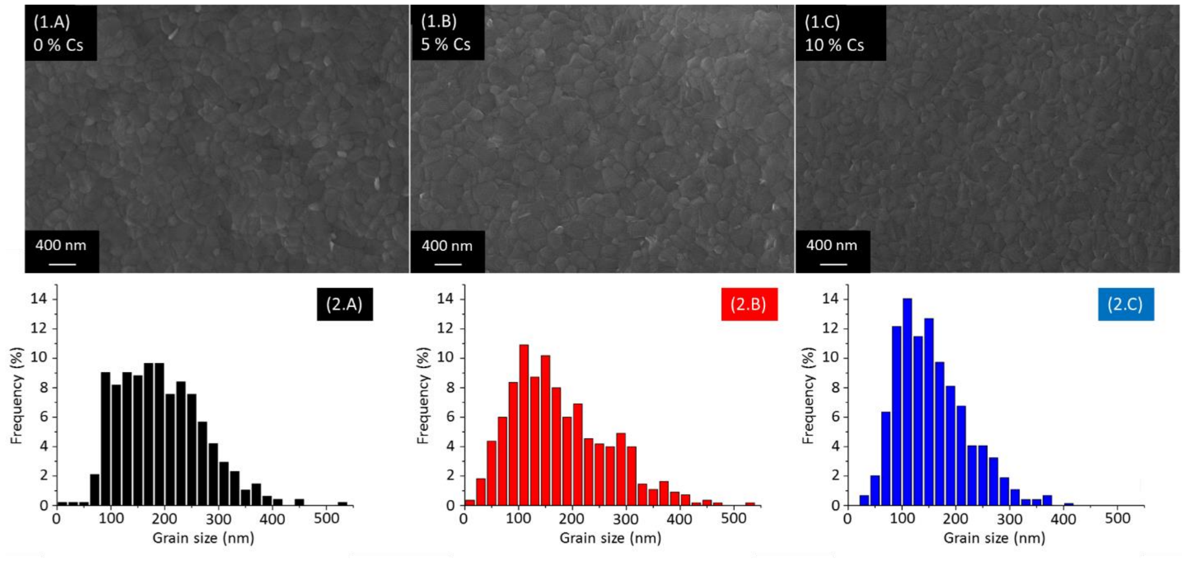

Figure S1. (1) Top-view Scanning Electron Microscopy (SEM) images (x50 k magnification) and (2) grain size distribution for the three different perovskite absorber films synthetized in this work: 0% Cs (A), 5% Cs (B) and 10% Cs (C).

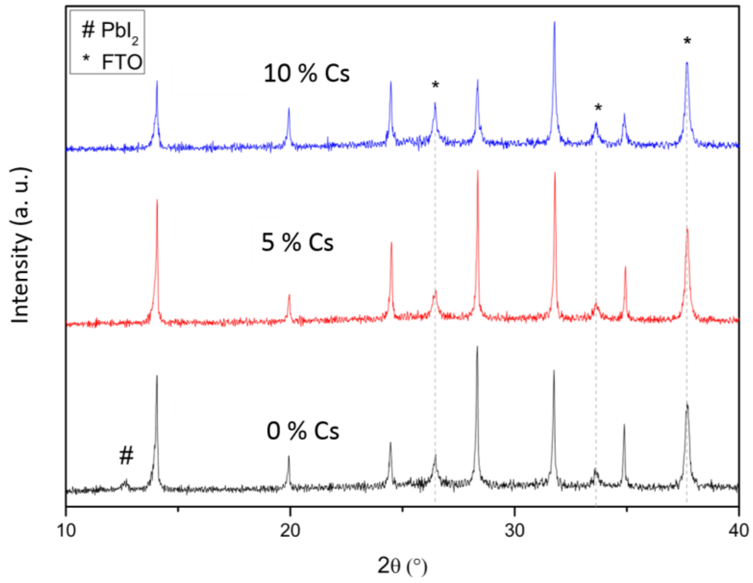

Figure S2. X-ray diffraction (XRD) diagrams of perovskite films grown onto FTO substrates with different caesium content (0% Cs, 5% Cs and 10 % Cs).

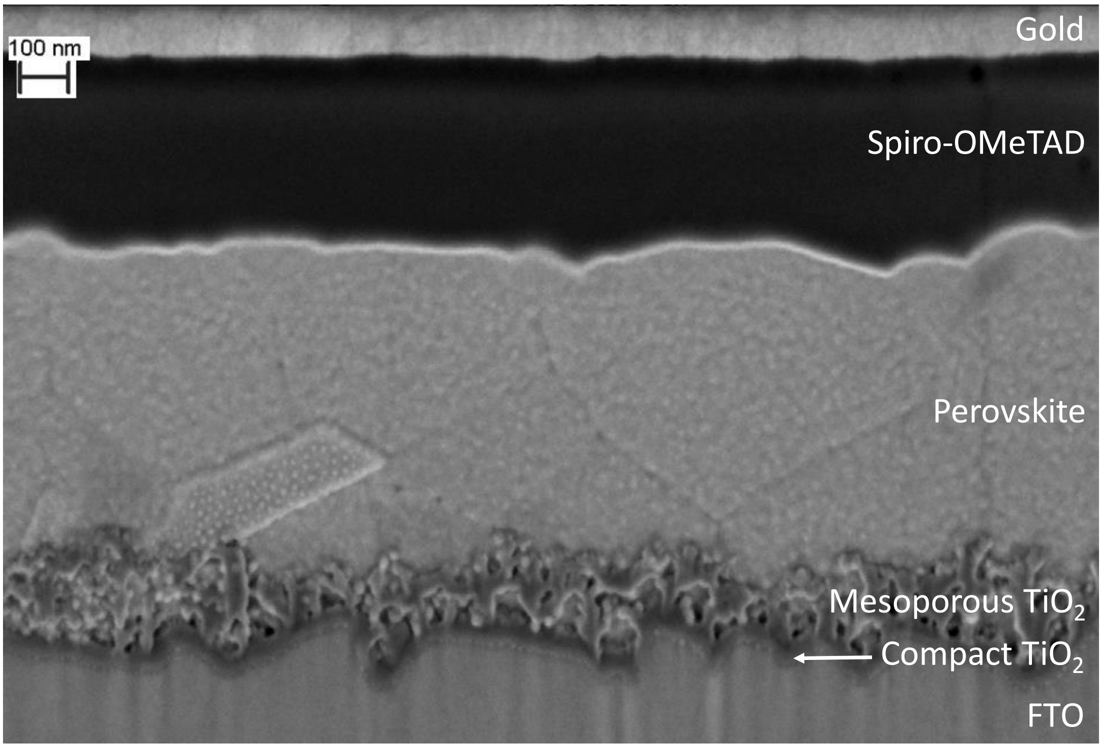

Figure S3. Cross section image of a complete opaque perovskite solar cell using a 5% Cs content, made by Focused Ion Beam (FIB) polishing and Scanning Electron Microscopy (SEM) imaging.

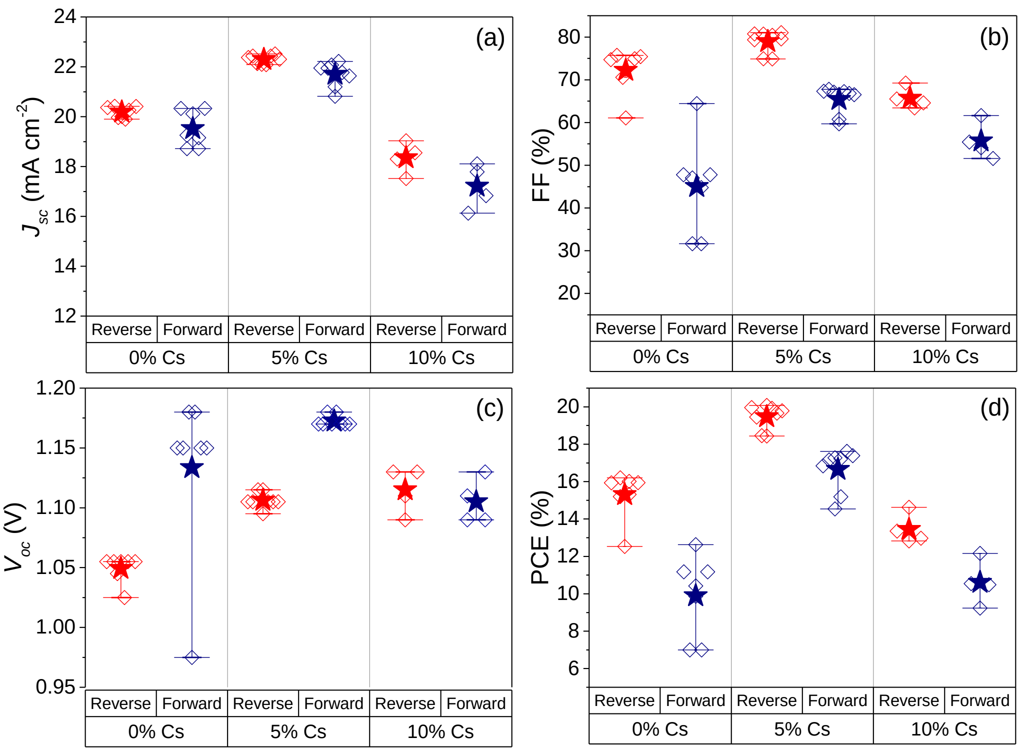

Figure S4. Photovoltaic parameters measured on opaque cells as the function of caesium content of the precursor solution. **(a)** Short circuit current density, **(b)** fill factor, **(c)** open circuit voltage and **(d)** power conversion efficiency. Mean values are shown as stars while whiskers represent the highest and lowest values.

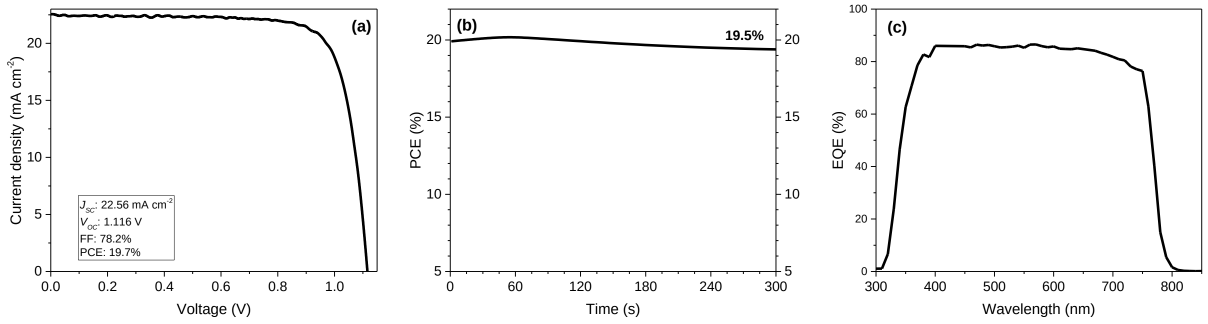

Figure S5. Photovoltaic characterization for the best-performing opaque PSC. **(a)** *J-V* characterization; scan-rate 80 mV s^{-1}. **(b)** Steady-state efficiency of champion opaque PSC at V_{MPP}. **(c)** External Quantum Efficiency (EQE).

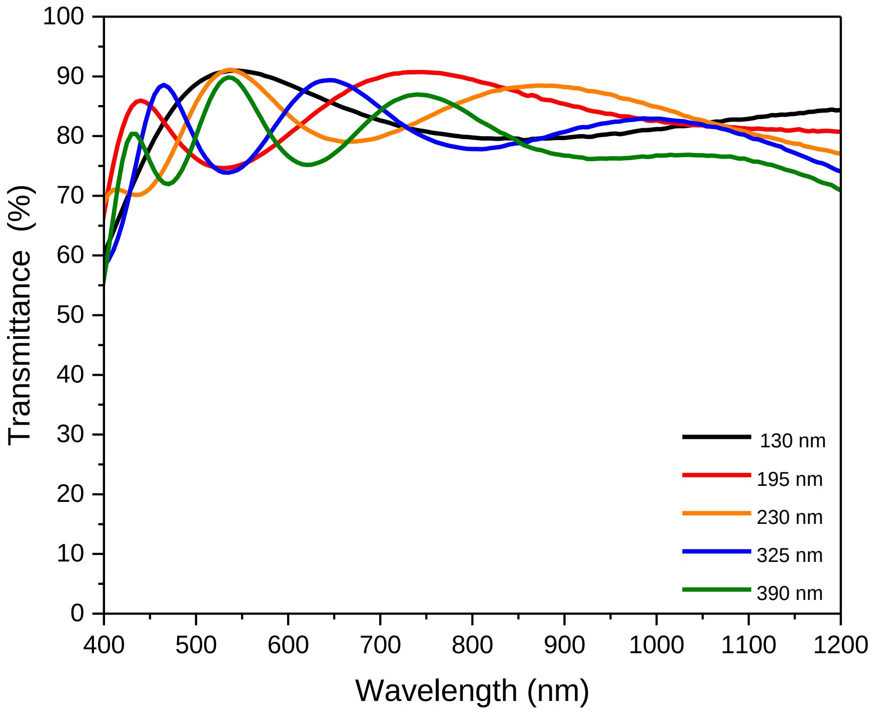

Figure S6. Transmission spectra of radio frequency (RF) magnetron sputtered ITO films with different thicknesses deposited onto borosilicate glass substrates.

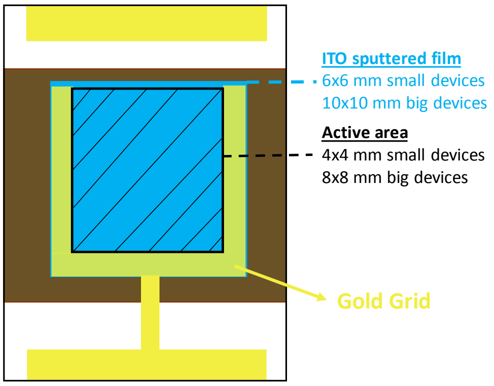

Figure S7. Schematic representation of the semitransparent PSC indicating the differences in size between small and big devices.

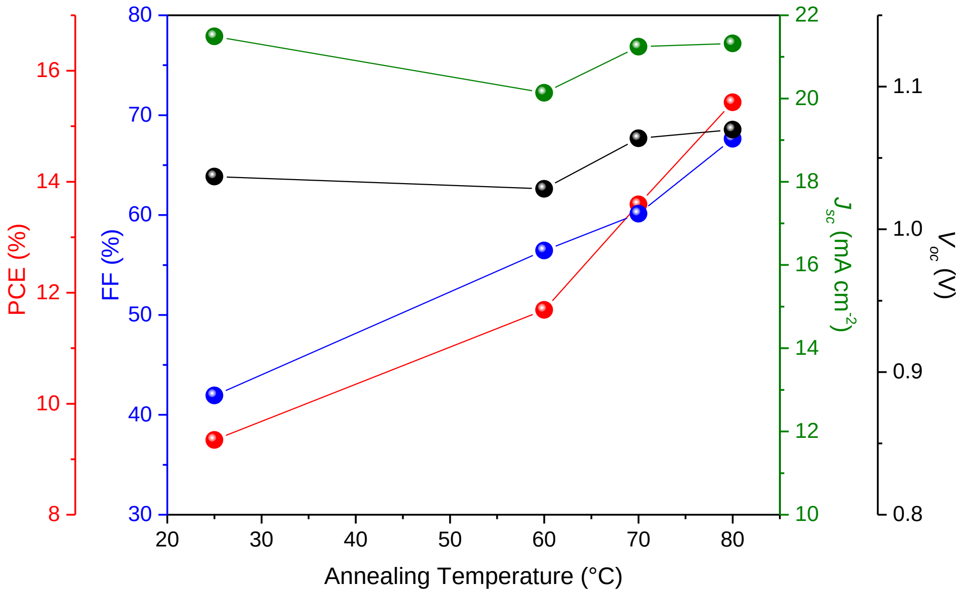

Figure S8. Evolution of photovoltaic parameters: J_{SC}, V_{OC}, FF and PCE extracted from *J*-*V* characterization for 0.64 cm^2 semitransparent PSC as a function of the annealing temperature.

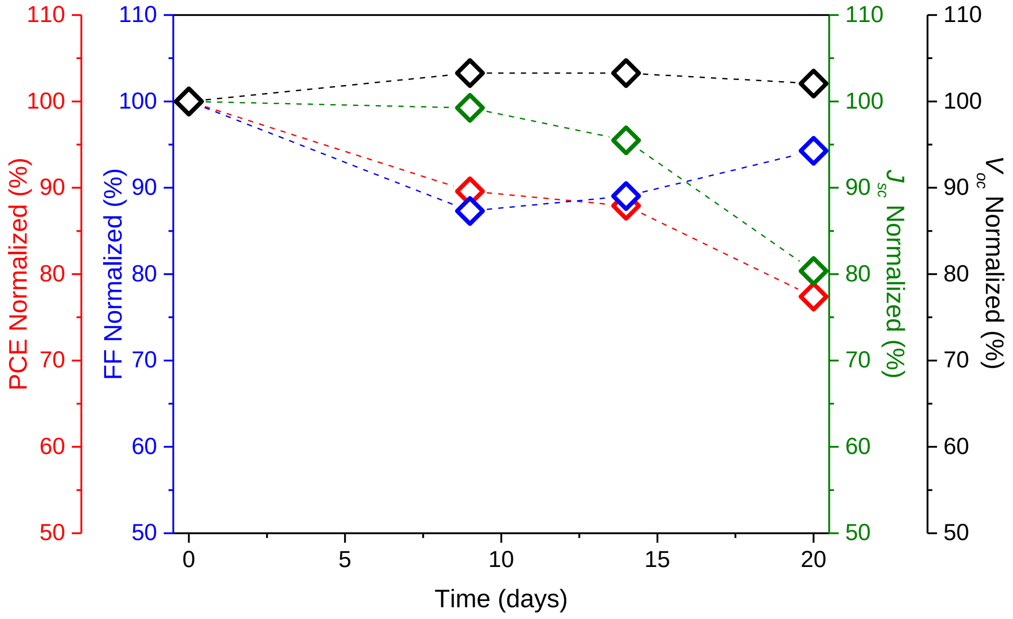

Figure S9. Evolution of photovoltaic parameters: J_{SC}, V_{OC}, FF and PCE (normalized) extracted from *J*-*V* characterization for the best-performing 0.64 cm^2 PSC as a function of time. Samples were stored in dark conditions between measurements.

TRPL model

The whole transport of charge carriers under pulsed excitation regime at open-circuit can be reproduced by writing a simple rate equation for the electron population (E1), with spatial Von Neumann boundary conditions (E2) and (E3). Where R_{pop} is the trap capture rate, R_{dep} is trap depopulation rate, S_{front} and S_{back} are recombination velocities linked to front and back interface (defined from the light incoming path), D_n is the electron diffusion coefficient, N_T is the shallow trap concentration, $n_{T,0}$ is the filled trap concentration during the decay.

$$\frac{\partial n}{\partial t} = -R_{eh}^{*} n\left(n_{T,0} + n\right) - R_{pop}\, n\left(N_T - n_{T,0}\right) + D_n \frac{\partial^2 n}{\partial z^2} \ (E1)$$
$$\forall t, z = 0 \rightarrow D_n \frac{\partial n}{\partial z} = S_{front}\, n \ (E2)$$
$$\forall t, z = z_0 \rightarrow D_n \frac{\partial n}{\partial z} = -S_{back}\, n \ (E3)$$

The temporal initial condition is determined by the absorption properties of the sample, before any in-depth diffusion or front surface recombination can happen. Where α_{532} is the absorption coefficient at the excitation wavelength.

$$t = 0 \rightarrow n = \Phi_0 \alpha_{532} \exp(-\alpha_{532} z) \ (E4)$$

Once this differential system solved, the PL intensity I_{PL} remains to be calculated. For the considered intrinsic semi-conductor where partial reabsorption of the PL is considered, it can be expressed as E5. Where α_{PL} is the average absorption coefficient at the emitted photoluminescence wavelength.

$$I_{PL} \propto \int_0^{z_0} R_{eh}^{*} n\left(n_{T,0} + n\right) \exp(-\alpha_{PL} z)\, dz \ \ (E5)$$

Then, classical optoelectronic properties such as the lifetime τ_n and the diffusion length L_n can be derived from the transport model described thanks to the following procedure. If one starts from the simple formula $L_n=\sqrt{(D_n\tau_n)}$, a difficulty quickly arises as τ_n is strongly injection-dependent for an intrinsic semi-conductor with a bimolecular recombination regime. Here, we determine the charge carrier concentration without the features linked to the pulsed excitation regime (*i.e.* in-depth diffusion can be neglected and shallow traps dynamics should be reconsidered. It corresponds to (E6-E8)[1] which we solve under a steady state illumination, using the fitted R_{eh}* and N_T coefficients.

$$\frac{\partial n}{\partial t} = -R_{eh} np - R_{pop}\, n(N_T - n_T)(E6)$$
$$\frac{\partial n_T}{\partial t} = R_{pop}\, n(N_T - n_T) - R_{dep}\, n_T p \ (E7)$$
$$\frac{\partial p}{\partial t} = -R_{eh} np - R_{dep}\, n_T p \ (E8)$$

It converges to a unique stable solution, giving access to the steady state filled trap concentration n_{T1sun} and electron concentration n_{1sun}. Interestingly, both increase significantly when N_T grows. Thereafter, τ_{1sun} and L_{1sun} can be calculated thanks to formulas (E7-8). Though trap depopulation plays a role in steady state condition, it is considered as n_{T1sun}<N_T and hence only R_{pop} and R^*_{eh} appear in this formula.

$$\tau_{1sun} = \frac{n_{1sun}}{R_{1sun}} = \frac{1}{R_{pop}\left(N_T - n_{T,1sun}\right) + R_{eh}^{*}\left(n_{1sun} + n_{T,1sun}\right)} \ (E9)$$
$$L_{1sun} = \sqrt{D_n \tau_{1sun}} \ (E10)$$

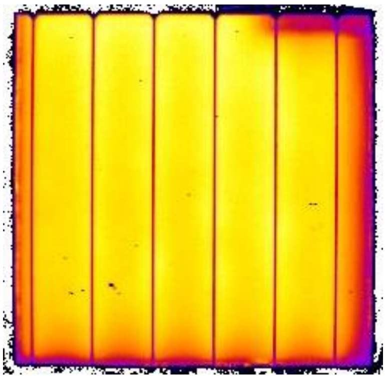

Figure S10. Electroluminescence measurement of the Al-BSF silicon solar cell employed as bottom cell in this work after laser cutting from Al-BSF silicon wafer.

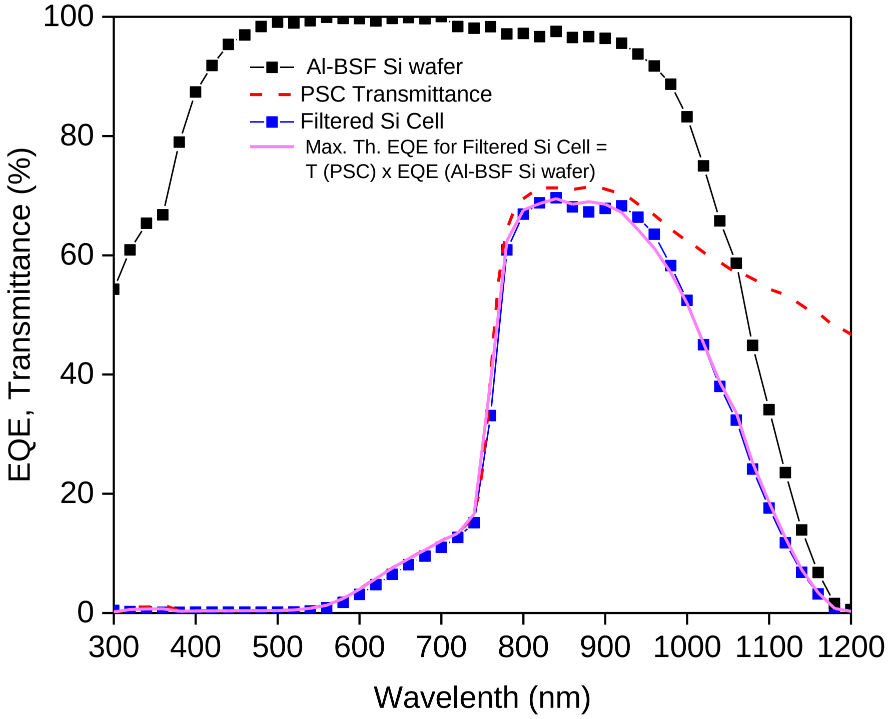

Figure S11. External Quantum Efficiency (EQE) measurements of the filtered silicon solar cell used as bottom cell (blue line with blue squares) compared with theoretical maximum EQE for filtered silicon (pink line). Max. Th. EQE (filtered Si) = *T* (PSC) x EQE (Al-BSF Si wafer). EQE from Al-BSF wafer (black line with black squares) and transmittance spectra of semitransparent PSC (dashed red line).

References

1. Stranks, S. D. *et al.* Recombination Kinetics in Organic-Inorganic Perovskites: Excitons, Free Charge, and Subgap States. *Phys. Rev. Appl.* **2**, 34007 (2014).
